# DeepTrio: a ternary prediction system for protein–protein interaction using mask multiple parallel convolutional neural networks

**DOI:** 10.1093/bioinformatics/btab737

**Published:** 2021-10-25

**Authors:** Xiaotian Hu, Cong Feng, Yincong Zhou, Andrew Harrison, Ming Chen

**Affiliations:** Department of Bioinformatics, College of Life Sciences, Zhejiang University, Hangzhou 310058, China; Department of Bioinformatics, College of Life Sciences, Zhejiang University, Hangzhou 310058, China; Department of Bioinformatics, College of Life Sciences, Zhejiang University, Hangzhou 310058, China; Department of Mathematical Sciences, University of Essex, Colchester CO4 3SQ, UK; Department of Bioinformatics, College of Life Sciences, Zhejiang University, Hangzhou 310058, China; Biomedical Big Data Center, the First Affiliated Hospital, Zhejiang University School of Medicine; Institute of Hematology, Zhejiang University, Hangzhou 310058, China

## Abstract

**Motivation:**

Protein–protein interaction (PPI), as a relative property, is determined by two binding proteins, which brings a great challenge to design an expert model with an unbiased learning architecture and a superior generalization performance. Additionally, few efforts have been made to allow PPI predictors to discriminate between relative properties and intrinsic properties.

**Results:**

We present a sequence-based approach, DeepTrio, for PPI prediction using mask multiple parallel convolutional neural networks. Experimental evaluations show that DeepTrio achieves a better performance over several state-of-the-art methods in terms of various quality metrics. Besides, DeepTrio is extended to provide additional insights into the contribution of each input neuron to the prediction results.

**Availability and implementation:**

We provide an online application at http://bis.zju.edu.cn/deeptrio. The DeepTrio models and training data are deposited at https://github.com/huxiaoti/deeptrio.git.

**Supplementary information:**

Supplementary data are available at *Bioinformatics* online.

## 1 Introduction

Various kinds of biological macromolecule interactions, especially protein–protein interactions (PPIs) (Jones and Thornton, 1996), play a fundamental role in biological information exchange, energy production and material transportation. A number of high-throughput and low-throughput experimental approaches, like yeast-two-hybrid purification followed by mass spectrometry (Lage, 2014), affinity capture-western, cocrystal structure analysis, bimolecular fluorescence complementation and biochemical modification analysis (Oughtred *et al.*, 2019), have been leveraged to identify PPIs. Thus, a tremendous number of PPIs have been identified and used to construct PPI databases, such as DIP (Salwinski *et al.*, 2004; Xenarios *et al.*, 2002), BioGRID (Oughtred *et al.*, 2019; Stark *et al.*, 2006) and STRING (Szklarczyk *et al.*, 2019), which makes it possible to identify PPIs in silico instead of the time-consuming and labor-intensive experimental methods.

Traditionally, protein 3D structure has been regarded as an essential profile for PPI prediction. However, with the discovery of intrinsically disordered proteins whose spatial structures interconvert on a series of timescales (Uversky *et al.*, 2008), the protein 3D structure is no longer regarded as the only determinant of PPIs, and that the protein primary structure may offer more clues for PPI prediction. Since the protein sequence can be easily obtained by many inexpensive and time-saving experimental technologies or directly inferred from gene sequences, it has become the most accessible type of protein profiles. Currently, a variety of protein properties can be predicted using the protein sequences. Some of them only depend on the protein itself like solubility (intrinsic property), while others require the information from another object like PPI (relative property). However, there are few existing prediction methods consider PPI as a relative property.

Many sequence-based machine learning methods have been developed for PPI prediction, such as Guo’s work (Guo *et al.,* 2008), Wang’s work (Wang *et al.*, 2018), DPPI (Hashemifar *et al.*, 2018), DNN-PPI (Li *et al.*, 2018), DeepFE-PPI (Yao *et al.*, 2019) and Protein–Protein Interaction Prediction Based on Siamese Residual RCNN (PIPR) (Chen *et al.*, 2019). Guo’s work (Guo *et al.*, 2008) curates seven physicochemical properties of amino acids (such as hydrophobicity, polarity and volumes of side chains) as protein feature descriptors. Each protein sequence is represented as seven vectors according to these descriptors. For a given protein sequence, auto covariance (AC) variables are used to describe the average interactions between residues throughout the whole sequence, and in downstream analysis, a support vector machine (SVM) (Cortes and Vapnik, 1995) is leveraged to determine whether the given proteins interact. DPPI (Hashemifar *et al.*, 2018) utilizes PSI-BLAST (Altschul *et al.*, 1997) to construct a comprehensive protein representation. DPPI incorporates a random projection module into the convolutional neural network (CNN) architecture, which projects the protein representations learned by the convolutional layers to two different vector spaces. The random projection module can help the model learn about the interaction potential of two input proteins. Finally, a linear transformation unit computes a probability value indicating whether two proteins interact in the prediction module. DeepFE-PPI (Yao *et al.*, 2019) exploits a novel residue representation method, Res2vec, to embed protein sequences, which may describe more precisely residue–residue interactions and supply more effective information for the downstream model. DeepFE-PPI employs the deep neural networks (DNN) as the learning architecture, and uses both a batch normalization module and a dropout module to prevent over-fitting. PIPR (Chen *et al.*, 2019) uses a pretrained semilatent vector to represent amino acids for capturing their contextual similarity and physicochemical properties. PIPR employs a residual recurrent convolutional neural network (RCNN) as the model architecture, and achieves the state-of-the-art performance for PPI prediction. In addition, PIPR is extended to contain three independent models for different application scenarios involving PPI prediction, interaction type prediction and binding affinity estimation.

Although a growing number of PPI predictors have been proposed in recent years, there remains some room for improvement: (i) it can be beneficial for prediction if a model can consider PPI as a relative property rather than an intrinsic property; (ii) few efforts have been made to provide an intuitive description of the inner mechanism of pairwise-input neural networks and illustrate the effect of each amino acid residue on PPI.

In this paper, we propose DeepTrio, a deep-learning framework based on a mask multiscale CNN architecture, in which multiple parallel filters provide valuable insights for PPI prediction by apprehending the multiscale contextual information of protein sequences. In comparison to existing tools, the main contributions of our work are: (i) an additional class, single-protein class, is introduced to our model, which allows DeepTrio to discriminate between the relative property and intrinsic property; (ii) due to the application of the single-protein class and masking operation, DeepTrio requires only one training set to build a model that can not only identify PPIs, but also further investigate the effect of each protein residue on PPI without any additional specific training; (iii) DeepTrio is also available as an online tool for inexperienced users in order to address the cross-platform usage and dependency related issues.

## 2 Materials and methods

**Fig. 1.**
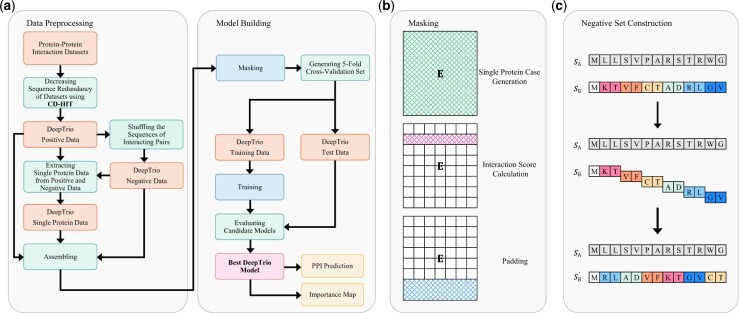
Details of the DeepTrio framework. (**a**) The development flowchart of DeepTrio. (**b**) Masking operation for three different purposes: generating single-protein cases, calculating the effect of each residue on PPI and padding the short sequences. (**c**) The strategy for constructing BioGRID negative datasets. Given an interacting protein pair $S_{A}$ and $S_{B}$, we randomly choose one protein (e.g. $S_{B}$) from them, and then shuffle its sequence with 2-let counts (excluding the first residue) to get a novel protein $S_{B}^{\prime }$. A negative sample is generated by pairing $S_{A}$ and $S_{B}^{\prime }$

Since PPI prediction is a binary classification task, most of the existing models are trained to classify the input data into two classes: interacting or noninteracting. However, we have designed DeepTrio for ternary prediction that takes as input a pair of protein sequences, and generates a three-dimensional vector output indicating the probability of interaction, noninteraction and single-protein. The overall framework of DeepTrio is illustrated in Figure  1a. DeepTrio also employs a Siamese architecture, which involves two identical subnetworks sharing the same configuration and weights, to ensure that two input sequences are represented and analyzed equally. In addition, DeepTrio can calculate the importance score for each residue by using the masking method.

### 2.1 Data collection

There are four datasets used for training and testing the models in this study. Two datasets are derived from the Biological General Repository for Interaction Datasets (BioGRID) (Oughtred *et al.*, 2019), and the other two datasets are derived from the database of interacting proteins (DIP) (Salwinski *et al.*, 2004; Xenarios *et al.*, 2002).

#### 2.1.1 BioGRID multivalidated physical interaction data

The BioGRID database (Oughtred *et al.*, 2019) is a comprehensive, specialized database for PPIs derived from multiple major species, whose multivalidated physical interaction subsets curate PPIs according to the criteria by which the interacting pairs must be validated in at least two different experimental systems or two different publication sources. Since the *Saccharomyces cerevisiae* (yeast) and *Homo sapiens* (human) data are widely used to evaluate the performance of PPI predictors (Chen *et al.*, 2019; Guo *et al.*, 2008; Hashemifar *et al.*, 2018; Yao *et al.*, 2019), we use the human and yeast multivalidated physical interaction datasets in BioGRID as the benchmarks for training and evaluating. The protein sequences are retrieved from the UniProt (UniProt Consortium, 2019) and restricted in length to a minimum of 150 and a maximum of 1500 residues. The human dataset involves 7705 proteins forming 31 164 positive cases and the yeast dataset contains 3553 proteins forming 13 462 positive cases. Following the same strategy as PIPR, we use CD-HIT (Fu *et al.*, 2012; Li and Godzik, 2006) to decrease sequence redundancy of the datasets, in which two PPIs are considered similar if they share a sequence identity greater than 40%.

The negative samples in these two benchmarks are generated by shuffling one sequence of a positive case with 2-let counts (excluding the first residue of the protein) (Fig.  1c). It has been demonstrated that the possibility of interaction can be deemed negligible if a sequence of one interacting pair is shuffled (Kandel *et al.*, 1996). Additionally, the shuffled sequence retains the same amino acid composition and approximately the same di-peptide frequencies as the original sequence.

#### 2.1.2 *Saccharomyces cerevisiae* core data

The *S.cerevisiae* core dataset, as a widely used benchmark, is composed of 11 188 PPI cases including 5594 positive cases proposed by Guo *et al.* (2008) and a heterogeneous set of 5594 negative cases according to different papers. The positive cases are selected from the DIP database (Salwinski *et al.*, 2004; Xenarios *et al.*, 2002), where proteins shorter than 50 amino acids and sharing ≥40% sequence identity are removed. The negative cases in these datasets are generated by randomly pairing the proteins without obvious evidence of interaction. However, there are some differences between the *S.cerevisiae* positive sets from DeepFE-PPI and PIPR, so we use both of the *S.cerevisiae* datasets to train and test DeepTrio and other baseline approaches.

#### 2.1.3 Single-protein data

The single-protein case consists of two components: a normal protein sequence and a masked sequence whose all residues are masked by blank bits (Fig.  1b). Each unique sequence in the positive and negative datasets corresponds to one case in the single-protein set. This set is designed for relieving the obscure influence caused by the relative property and preventing the potential weight polarization in the intermediary layers. The way we train single-protein data are the same as the positive and negative cases. Note that this set is only used for training DeepTrio, and does not participate in the evaluation for DeepTrio.

### 2.2 Protein feature encoder

**Fig. 2.**
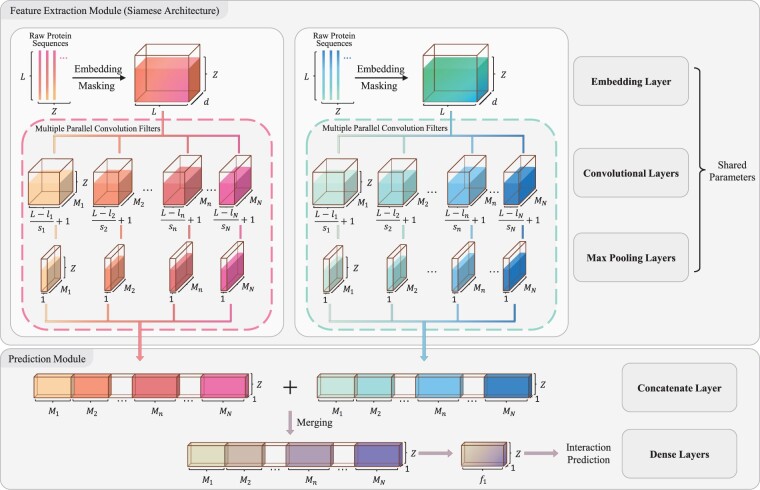
DeepTrio overall architecture. The protein sequences are converted into liquid-like tensors by a tunable embedding module. The container-like layers of different size constantly shape the flowing tensors, where Z is the number of samples in a batch

DeepTrio employs a Siamese architecture with the multiple parallel convolution (multiscale convolution) module to capture various protein features in multiscale windows. It takes as input a protein pair (X, $X^{\prime }$**)**, and yields two protein representations ($H_{\mathrm{conc}.},\;{H'}_{\mathrm{conc}.}$) for downstream analysis (Fig.  2).

#### 2.2.1 Single-protein data

The input protein sequence is projected into a sparse orthonormal vector space by performing one-hot encoding transformation in the input module. For two input proteins $S_{A}$ and $S_{B}$, each of them is transformed into a binary matrix $X\in R^{L\times 23}$ as follows:

X= x1⋮ xi⋮ xL,

where $x_{i}\in R^{1\times 23}$ (i=1, 2,…,L) is a binary vector of length 23 (22 for the proteinogenic amino acids and 1 for the mask bit) corresponding to the $i_{\mathrm{th}}$ amino acid residue in a sequence, and L is fixed to 1500. A trainable embedding weight matrix $W_{e}\in R^{23\times d}$ (optimized by backpropagation) is used to map X to a dense continuous vector space by the following equation:

E=XWe,

where $E\in R^{L\times d}$ is the embedded representation of one input protein and d is the feature dimension of the amino acid symbol lexicon.

#### 2.2.2 Masking module

A Boolean matrix, $B\in R^{L\times 1}$, will be attached to the embedded representation E in this module, which eliminates the masked residues from the calculation in the downstream modules. This operation will be called in three scenarios (Fig.  1b):

- The length of protein sequences is fixed to 1500. Thus, the shorter sequences will be padded with mask bits.
- In the single-protein case, the whole sequence of one of the proteins is masked by mask bits. Thus, there is only one protein participating in the calculation of the deep-learning model when the single-protein case is inputted.
- When DeepTrio investigates the effect of a particular residue $b_{i}$ on PPI, a mask bit will be attached to this residue, which blocks the calculation of $b_{i}$ in the downstream layers.

#### 2.2.3 Multiple parallel convolutional module with pooling

The embedded representation E is analyzed by N parallel convolution filters with $M_{n}$ (n=1, 2,…,N) kernels (Fig.  2). Each convolution filter extracts a certain specific aspect of protein profiles and outputs as follows:

Tk,mn=∑i=1ln∑j=1dvi,j(m,n)×Ei+k-1×sn,j,

where $l_{n}$ and $s_{n}$ denote the length of the convolution window and stride in the $n_{\mathrm{th}}$ convolution filter, respectively. The output $T_{k,m}^{\left(n\right)}$ ($k=1,\;2,\ldots ,\frac{L-l_{n}}{s_{n}}+1$) is the $m_{\mathrm{th}}$ interior element in the $k_{\mathrm{th}}$ row of the $n_{\mathrm{th}}$ convolution filter, $v_{i,j}^{\left(m,n\right)}$ is the $j_{\mathrm{th}}$ interior element in $i_{\mathrm{th}}$ row of the $m_{\mathrm{th}}$ kernel in the $n_{\mathrm{th}}$ convolution filter, and $E_{i+k,j}$ is the $j_{\mathrm{th}}$ interior element in ${\left(i+k\right)}_{\mathrm{th}}$ row of the embedded matrix E. Note that the bias calculation is not applied to the convolution calculation.

The filter outputs are activated by the rectified linear unit (ReLU) (Xu *et al.*, 2015) and yield a set of feature maps, $\left\{A^{\left(n\right)}\in R^{\left(\frac{L-l_{n}}{s_{n}}+1\right)\times M_{n}},n=1,\;2,\ldots ,N\right\}$, which are calculated as follows:

Ak,mn=ReLUTk,mn,

where $A_{k,m}^{\left(n\right)}$ is the $m_{\mathrm{th}}$ interior element in the $k_{\mathrm{th}}$ row of $A^{\left(n\right)}$. After obtaining these feature maps, a global max-pooling operation is performed for reducing the dimension of feature maps and highlighting the most significant features. The max-pooling output $H^{\left(n\right)}\in R^{1\times M_{n}}$ (for the $n_{\mathrm{th}}$ convolution filter) is given by

$$\begin{array}{c} h_{m}^{\left(n\right)}=\max\left(A_{1,m}^{\left(n\right)},A_{2,m}^{\left(n\right)},\ldots ,A_{\frac{L-l_{n}}{s_{n}}+1,m}^{\left(n\right)}\right), \end{array}$$

 

Hn=h1n,h2n,…,hmn,…,hMnn,

where $h_{m}^{\left(n\right)}$ is the $m_{\mathrm{th}}$ element of $H^{\left(n\right)}$. Next, we flatten and concatenate all the $H^{\left(n\right)}$ (n=1, 2,…, N) to get a new row vector $H_{\mathrm{conc}.}\in R^{1\times N}$:

Hconc.=H1;H2;…;HN.

### 2.3 Prediction and learning objectives

Two max-pooling outputs generated by the aforementioned modules are first merged into one vector, and then passed into the dense layers to calculate the probability value for PPI. The learning architecture is trained to optimize the cross-entropy loss between predictions and targets by backpropagation with AMSGrad algorithm (Reddi *et al*., 2019).

#### 2.3.1 Prediction module

Two max-pooling outputs, $H_{\mathrm{conc}.}^{A}$ and $H_{\mathrm{conc}.}^{B}$, given by the two subnetworks (sharing the same configuration and weights), are combined via element-wise addition and transformed into a merged vector $H_{\mathrm{merged}}\in R^{1\times N}$. Compared with the element-wise multiplication, the addition operation prevents $H_{\mathrm{merged}}$ being a zero-vector when the single-protein case is inputted. The merged vector $H_{\mathrm{merged}}$ is first passed through two dense layers, and then normalized by the softmax function as follows:

F=ReLUReLUHmergedWf1Wf2,

 

ci=σFi=exp⁡Fi∑j=13exp⁡Fj,

where $W_{f_{1}}\in R^{N\times f_{1}}$, $W_{f_{2}}\in R^{f_{1}\times 3}$ are the weight matrices of the first and the second dense layers, respectively. The $i_{\mathrm{th}}$ dimension of $c\in R^{1\times 3}$ corresponds to the confidence score, $c_{i}\in [0,1]$, of the $i_{\mathrm{th}}$ class.

#### 2.3.2 Learning objective

For a given protein pair p, its class label $y^{p}$ is defined as

yp=1,0,0 interacting 0,1,0 negative 0,0,1 single protein.

The learning model is trained to minimize the following cross-entropy loss and classify the inputs into their corresponding classes correctly

Loss=CEEcp,yp=-1Z∑z=1Z∑i=13yipln⁡c ip,

where CEE is the cross-entropy error function, $c_{i}^{p}$ and $y_{i}^{p}$ represent the $i_{\mathrm{th}}$ scalar components of the model prediction $c^{p}$ and its corresponding class label $y^{p}$, respectively, and Z is the number of inputs in a batch.

#### 2.3.3 Optimization strategy

We adopt AMSGrad (Reddi *et al.*, 2019), a variant of Adam optimizer (Kingma and Ba, 2014), to optimize the cross-entropy loss of our learning model. Following the same strategy as PIPR, the learning rate α is set to 0.001, and the exponential decay rates $\beta_{1}$ and $\beta_{2}$ are set to 0.9 and 0.999, respectively.

#### 2.3.4 Hyperparameter tuning

The hyperparameter searching space of our model consists of 13 dimensions (including the hyperparameters for the embedding dimension, dropout rates, convolution kernel lengths, convolution strides and optimizers), which form about 140 000 combinations (Supplementary Table S1). It is too large for the grid search algorithm to find the optimal combination. Therefore, we leverage a Bayesian tuning tool GpyOpt (The GPyOpt Authors, 2016) to optimize the search process, which has been proved to be more efficient than the randomized grid search (Wang *et al.*, 2019). For the optimization program GpyOpt, we set the number of initial random searching points and the maximum number of iterations to 10 and 50, respectively. The performance of all candidate models and their corresponding hyperparameter settings are listed in Supplementary Table S2.

#### 2.3.5 Implementation details

We randomly initialize the weights of the embedding, convolution and dense layers according to the Glorot uniform distribution (Glorot and Bengio, 2010), which is a common strategy used by deep-learning methods for model initialization (Kulmanov *et al.*, 2018; Seo *et al.*, 2018; Wang *et al.*, 2020). We design DeepTrio based on the open-source TensorFlow 2.0 library (Abadi *et al.*, 2016), and implement training and evaluation for all baseline models using a NVIDIA Tesla P100 GPU with 16 GB of memory.

### 2.4 Calculating the effect of protein residues on prediction

Suppose we have a pair of interacting proteins $S_{A}=(b_{1}^{A},b_{2}^{A},\ldots ,b_{i}^{A},\ldots ,b_{L}^{A})$ and $S_{B}=(b_{1}^{B},b_{2}^{B},\ldots ,b_{i}^{B},\ldots ,b_{L}^{B})$, where $b_{i}^{A}$ and $b_{i}^{B}$ are the $i_{\mathrm{th}}$ residues of $S_{A}$ and $S_{B}$, respectively. To calculate the effect of the residue $b_{i}^{A}$ on prediction with respect to $S_{B}$, we first calculate the probability that $S_{A}$ does not interact with $S_{B}$ [i.e. $P_{\mathrm{neg}.}(S_{A},S_{B})$]. Second, we attach a mask vector (with an inactive bit in the $i_{\mathrm{th}}$ component) to the embedded representation of $S_{A}$ (generating a new sequence called ${\hat{S}}_{A}$) and recalculate the probability that ${\hat{S}}_{A}$ does not interact with $S_{B}$ [i.e. $P_{\mathrm{neg}.}({\hat{S}}_{A},S_{B})$]. Finally, the effect of $b_{i}^{A}$ on prediction with respect to $S_{B}$ is assigned to be

UBbiA=Pneg.S^A,SB-Pneg.SA,SB.

## 3 Results

We report the performance of DeepTrio and other approaches on four different PPI datasets. Further, we test the performance of DeepTrio on the multiple specie dataset where proteins are filtered based on different thresholds of sequence identity. In addition to the binary prediction of PPIs, DeepTrio can generate an intuitive protein portrait for the detection of potentially important residues for interaction. Lastly, a logically concise online application has been developed to help researchers make better use of DeepTrio.

### 3.1 Performance comparison of DeepTrio with other approaches

The main task of DeepTrio is to estimate the interaction probability of a given protein pair based on its sequences. We compare DeepTrio with several state-of-the-art PPI prediction methods including SVM-AC (Guo *et al.*, 2008), SVM-MCD (You *et al.*, 2014), DPPI (Hashemifar *et al.*, 2018), PIPR (Chen *et al.*, 2019) and DeepFE-PPI (Yao *et al.*, 2019) on a variety of benchmark datasets. Furthermore, we also report the performance of a simplified variant of DeepTrio (named as DeepDuo), which has the same learning architecture as DeepTrio but is not trained by the single-protein dataset. By setting the simplified control of DeepTrio, we can further investigate how the single-protein cases influence the prediction performance of our model.

#### 3.1.1 BioGRID multivalidated physical interaction data

We perform 5-fold cross-validation on the BioGRID human and yeast datasets. Under this setting, the data are equally divided into five parts and each part has an equal chance to train and test the models. We aggregate eight quality metrics including accuracy, precision, sensitivity, specificity, F1 score, Matthews correlation coefficient (MCC) and average precision (AP) to assess the prediction performance of the models. Higher values in all these metrics indicate better performance.

As shown in Tables  1 and 2, the RCNN architecture of PIPR promises a remarkable performance and gets the highest scores in sensitivity on both the human and yeast datasets. However, DeepTrio achieves the best performance in other metrics by leveraging a multiscale convolution architecture that can better learn the deep features from protein sequences. For example, DeepTrio outperforms PIPR by 0.52% and 1.79% in accuracy, and by 1.43% and 4.34% in precision on the human and yeast datasets, respectively.

**Table 1.** Evaluation of PPI prediction performance on the BioGRID *S.cerevisiae* dataset based on 5-fold cross-validation

Methods	Accuracy (%)	Precision (%)	Sensitivity (%)	Specificity (%)	MCC (%)	F1-score (%)
DeepFE-PPI^a^ (Yao *et al.*, 2019)	85.24 ± 0.52	85.49 ± 1.41	84.99 ± 2.77	85.49 ± 2.11	70.57 ± 1.06	85.19 ± 0.79
PIPR^a^ (Chen *et al.*, 2019)	95.76 ± 0.25	94.61 ± 0.53	97.06 ± 0.41	94.47 ± 0.55	91.56 ± 0.48	95.82 ± 0.24
DeepDuo^a^	97.06 ± 0.28	98.06 ± 0.51	96.02 ± 0.35	98.10 ± 0.50	94.14 ± 0.57	97.02 ± 0.30
DeepTrio^a^	97.55 ± 0.38	98.95 ± 0.20	96.12 ± 0.74	98.98 ± 0.21	95.15 ± 0.74	97.52 ± 0.40

*Note*: We report the mean values and standard deviations for the test sets.

a Those models are retrained using the same data.

**Table 2.** Evaluation of PPI prediction performance on the BioGRID *H.sapiens* dataset based on 5-fold cross-validation

Methods	Accuracy (%)	Precision (%)	Sensitivity (%)	Specificity (%)	MCC (%)	F1-score (%)
DeepFE-PPI^a^ (Yao *et al.*, 2019)	87.66 ± 0.57	89.42 ± 1.05	85.47 ± 2.27	89.85 ± 1.40	75.44 ± 1.09	87.37 ± 0.78
PIPR^a^ (Chen *et al.*, 2019)	97.60 ± 0.08	97.57 ± 0.35	97.63 ± 0.44	97.56 ± 0.36	95.20 ± 0.15	97.60 ± 0.10
DeepDuo^a^	98.04 ± 0.05	98.83 ± 0.28	97.23 ± 0.28	98.85 ± 0.27	96.09 ± 0.10	98.02 ± 0.05
DeepTrio^a^	98.12 ± 0.12	99.00 ± 0.17	97.23 ± 0.28	99.01 ± 0.17	96.26 ± 0.23	98.11 ± 0.13

*Note*: We report the mean values and standard deviations for the test sets.

a Those models are retrained using the same data.

In addition, we report the comparison between DeepDuo and DeepTrio on the BioGRID benchmarks, which provides insights into the role of single-protein training in PPI prediction. It is observed that DeepTrio perform consistently better than DeepDuo in all of the evaluation metrics (Tables  1 and 2). For example, DeepTrio attains an accuracy value of 97.55% (which is 0.49% higher than DeepDuo), and an MCC value of 95.15% (which is 1.01% higher than DeepDuo) in the yeast dataset. These results suggest that the single-protein training process can improve our model performance on the BioGRID datasets.

#### 3.1.2 *Saccharomyces cerevisiae* core data

We first use DeepFE-PPI’s *S.cerevisiae* dataset to evaluate the performance of DeepTrio. The positive set from DeepFE-PPI is identical with that from You *et al.* (2015). To make the data suitable for the model input, we remove 255 cases that contains proteins longer than 1500 amino acids, and use the truncated data to retrain and evaluate DeepTrio and PIPR. The evaluation shows that, under the highest scores attained by DeepFE-PPI on its own data, DeepTrio achieves better performance than PIPR with respect to five evaluation metrics (Table  3). Second, we test the performance of DeepTrio and DeepFE-PPI on PIPR’s dataset, where we remove 231 cases containing proteins longer than 2000 amino acids. The results in Table  4 show that DeepTrio attains better performance than DeepFE-PPI (such as 3.74% higher in accuracy, 8.04% higher in precision and 7.44% higher in MCC) on PIPR’s dataset. However, PIPR achieves the state-of-the-art performance on its own dataset, but exhibits worse performance than DeepTrio in precision and specificity. In addition, DeepTrio also outperforms DeepDuo on both of the *S.cerevisiae* datasets in most metrics (Tables  3 and 4). Detailed performance of DeepTrio, PIPR and DeepFE-PPI on two *S* *cerevisiae* datasets is provided in the Supplementary Material.

**Table 3.** Evaluation of PPI prediction performance on the *S.cerevisiae* core dataset from DeepFE-PPI based on 5-fold cross-validation

Methods	Accuracy (%)	Precision (%)	Sensitivity (%)	Specificity (%)	MCC (%)	F1-score (%)
SVM-AC (Guo *et al.*, 2008)	87.35 ± 1.38	87.82 ± 4.84	87.30 ± 5.23	87.41 ± 6.33	87.34 ± 1.33	75.09 ± 2.51
SVM-MCD (You *et al.*, 2014)	91.36 ± 0.4	91.94 ± 0.69	90.67 ± 0.77	NA	91.3 ± 0.73	84.21 ± 0.59
DeepFE-PPI (Yao *et al.*, 2019)	94.78 ± 0.61	96.45 ± 0.87	92.99 ± 0.66	NA	NA	89.62 ± 1.23
DeepDuo^a^	92.16 ± 0.55	96.57 ± 1.22	87.46 ± 1.46	96.83 ± 1.27	91.78 ± 0.59	84.71 ± 1.10
PIPR^a^ (Chen *et al.*, 2019)	92.26 ± 0.44	94.17 ± 0.65	90.11 ± 0.56	94.42 ± 0.56	92.09 ± 0.53	84.60 ± 0.89
DeepTrio^a^	92.57 ± 0.63	96.33 ± 0.88	88.53 ± 1.19	96.62 ± 0.83	92.26 ± 0.65	85.43 ± 1.22

*Note*: Performance values for majority of baseline approaches are obtained from Yao *et al.* (2019), and NA denotes unavailability of the values from the original papers. We report the mean values and standard deviations for the test sets.

a Those models are retrained using the same data.

**Table 4.** Evaluation of PPI prediction performance on the *S.cerevisiae* core dataset from PIPR based on 5-fold cross-validation

Methods	Accuracy (%)	Precision (%)	Sensitivity (%)	Specificity (%)	MCC (%)	F1-score (%)
DPPI (Hashemifar *et al.*, 2018)	94.55	96.68	92.24	NA	94.41	NA
PIPR (Chen *et al.*, 2019)	97.09 ± 0.24	97.00 ± 0.65	97.17 ± 0.44	97.00 ± 0.67	97.09 ± 0.23	94.17 ± 0.48
DeepDuo^a^	94.14 ± 0.30	96.37 ± 1.43	91.74 ± 1.26	96.51 ± 1.41	93.98 ± 0.27	88.40 ± 0.67
DeepFE-PPI^a^ (Yao *et al.*, 2019)	91.04 ± 0.45	89.14 ± 1.58	93.52 ± 1.67	88.55 ± 2.01	91.25 ± 0.4	82.23 ± 0.86
DeepTrio^a^	94.78 ± 0.28	97.18 ± 0.28	92.20 ± 0.49	97.33 ± 0.30	94.63 ± 0.29	89.67 ± 0.55

*Note*: Performance values for majority of baseline approaches are obtained from Yao *et al.* (2019), and NA denotes unavailability of the values from the original papers. We report the mean values and standard deviations for the test sets.

a Those models are retrained using the same data.

#### 3.1.3 Comprehensive comparison between DeepTrio and PIPR

Based on the four datasets mentioned above, we count how many times DeepTrio or PIPR attains higher scores with respect to six metrics. Table  5 shows that DeepTrio offers robust performance over the four datasets and outperforms PIPR in many evaluation metrics, especially in precision and specificity.

**Table 5.** Statistics for the better performance achieved by DeepTrio and PIPR on four datasets with respect to six evaluation metrics

Methods	Accuracy	Precision	Sensitivity	Specificity	F1-score	MCC
PIPR	1	0	4	0	1	1
DeepTrio	3	4	0	4	3	3

### 3.2 PPI prediction on multispecies dataset

Following the same strategy as PIPR (Chen *et al.*, 2019), we perform 5-fold cross-validation of DeepTrio on the multispecies dataset (*Caenorhabditis elegans*, *Escherichia coli* and *Drosophila melanogaster*), where proteins are filtered based on different thresholds of sequence identity (40%, 25%, 10% and 1%). To make the data suitable for the model input, we also remove the cases containing proteins longer than 1500 amino acids. The results in Table  6 show that DeepTrio performs consistently well on a series of datasets with different sequence identities.

**Table 6.** Evaluation of PPI prediction performance on the multispecies (*C.elegans*, *D.melanogaster* and *E.coli*) dataset

Sequence identity	Protein number	Positive pairs	Negative pairs	Accuracy (%)	Precision (%)	Sensitivity (%)
Any	11 108	31 227	30 368	98.20	99.51	96.92
≤40%	9354	24 406	20 461	97.83	99.23	96.77
≤25%	7454	18 193	14 485	97.52	98.78	96.74
≤10%	5478	11 777	8839	97.32	98.87	96.42
≤1%	4932	10 110	7284	97.11	98.89	96.10

### 3.3 PPI prediction on independent test set

**Fig. 3.**
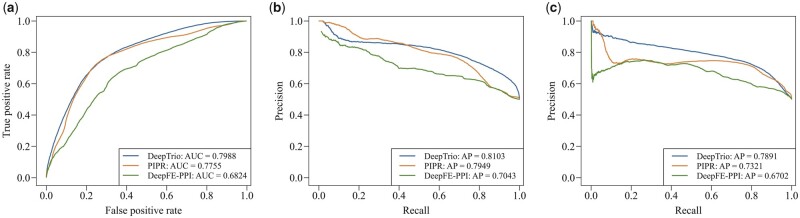
Performance comparison of DeepTrio with PIPR and DeepFE-PPI on independent test set. (**a**) Comparison of area under receiver operating curve (AUC). (**b**) Comparison of AP with respect to the interacting class. (**c**) Comparison of AP with respect to the noninteracting class

Here, we use the virus–human interaction dataset in Liu-Wei *et al.* (2021) as an independent test set to assess the performance of DeepTrio and other approaches (trained by the BioGRID human–human interaction dataset). Following the preprocessing methods in the previous studies (Hashemifar *et al.*, 2018; Khurana *et al.*, 2018; Rawi *et al.*, 2018), we first decrease sequence redundancy in the virus protein data with a maximum sequence identity of 10%. Second, we exclude all the virus sequences in the independent test set with a sequence identity of ≥25% to any sequence in the human–human interaction training set. The negative independent test data are generated by randomly shuffling the protein sequences in the virus–human interaction dataset (this method is elaborated in Section 2.1.1). The final independent test set is composed of 8929 interacting and 8929 noninteracting protein pairs. The results in Figure  3 show that DeepTrio exhibits competitive performance on the independent test set in comparison to PIPR.

### 3.4 Detecting and visualizing potentially important residues for interaction

Since experiment-based methods require meticulous operations and lots of time to identify the important sites for interaction, it is crucial to conduct a prior assessment of experimental protocols and prereject initial targets with the lowest interaction probability. Thus, we extend DeepTrio to an additional scenario that helps detect the potentially important sites for interaction (which are not limited to the residues in core binding regions, but also include some other crucial residues that shape the external and internal structures, provide skeleton support through long aliphatic side chains or create the hydrophobic environment). The main goal of this extension is to find out which residues take the main responsibility for the prediction results and visualize the importance score for each residue in a sequence.

Recently, a handful of previous works have already applied several visualization techniques to provide interpretable explanations for deep-learning models. DeepBind (Alipanahi *et al.*, 2015) uses ‘*mutation maps*’ to illustrate the effect that each possible point mutation may have on binding affinity between DNA and proteins. DeepChrome (Singh *et al.*, 2016) utilizes a network-centric approach (Yosinski *et al.*, 2015) to extract the class-specific feature patterns that are highly influential in gene expression predictions. DeepSig (Savojardo *et al.*, 2018) employs the deep Taylor decomposition approach (Montavon *et al.*, 2017) to determine a relevance score measuring the contribution of each input neuron toward the prediction. In this work, owing to the integration of the single-protein training strategy and masking operation, it is possible to allow DeepTrio to visualize the contribution of each input neuron toward the prediction (which is elaborated in Section 2.4).

**Fig. 4.**
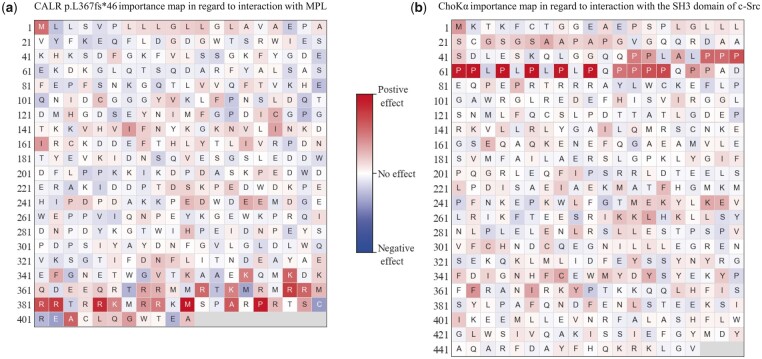
An ‘*importance map*’ are employed to visualize the effect of each amino acid residue on interaction, where residues in red colors exert positive effects and those in blue colors exert negative effects on prediction. (**a**) Analysis of the potential importance of each residue in CALR p. L367fs*46 for interaction with MPL. The positively charged residues in the last 36 amino acids exhibit a strong trend of higher importance scores, which have been proved essential for the physical interaction between CALR p. L367fs*46 and MPL. (**b**) Analysis of the potential importance of each residue in ChoKα for interaction with the SH3 domain of c-Src. The poly-proline region in ChoKα residues 53–78 harbors relatively higher scores in the *importance map*, which are reported crucial for the interaction with the SH3 domain of c-Src (Kall *et al.*, 2019)

We validate the visualization results given by DeepTrio (the model is trained using the BioGRID human multivalidated physical interaction data) with the recent experimental evidence in biochemical studies. Note that all the PPIs mentioned below, along with their mutants, are not included in the training data of DeepTrio. Figure  4a shows the *importance map* of the mutant human calreticulin (CALR) (that loses most of the C-terminal acidic residues and gains a novel common C-terminus with 36 amino acids rich in positively electrostatic charges caused by a heterogeneous set of +1 bp frameshift mutations in exon 9) (Nangalia *et al.*, 2013). These positively charged residues in the novel C-terminus are reported essential for mediating the erroneous activation of MPL signaling and the physical interaction between mutant CALR and the thrombopoietin receptor MPL, which can lead to myeloproliferative disorders (Elf *et al.*, 2016, 2018). We use the ‘*importance map*’ to illustrate the importance score of each residue in the mutant CALR (p.L367fs*46) (Fig.  4a). The ‘*importance map*’ is rendered as a heat map with *l* squares (where *l* is the length of the given protein), and each line in the heat map is set to 20 squares. It can be observed in Figure  4a that most of the residues with crimson backgrounds are enriched in the C-terminus, where the positively charged residues (like arginine and lysine) exhibit a strong trend of higher importance scores. These results are basically consistent with the previous findings in experimental studies (Elf *et al.*, 2016, 2018). Figure  4b depicts the *importance map* of Choline kinase alpha (ChoKα). ChoKα catalyzes the phosphorylation of choline to phosphocholine, and its high expression has proven to be associated with cancer malignancy and poor patient prognosis (Ramírez De Molina *et al.*, 2002, 2005). Recent biophysical and biochemical studies (Kall *et al.*, 2019) have demonstrated that the ChoKα poly-proline region in residues 49–79 (especially prolines 61 and 62) mediates the physical interaction between ChoKα and the SH3 domain of c-Src tyrosine kinases. It can be seen in the ChoKα *importance map* (Fig.  4b) that the highly scored residues are enriched in the N-terminal poly-proline region, which is consistent with the findings in the aforementioned experimental studies.

In practice, the *importance map* shows a preference for finding the key residues that share similar properties in the adjacent regions and a sensitivity decrease for large protein assessment. Another noteworthy observation in both Figures  4a and 4b is that the vast majority of the negative-effect residues harbor the pale-blue backgrounds, which can be explained by the hypothesis that most of point mutations will reduce the interaction between two proteins that have already reached the optimal conformation for binding.

### 3.5 Online server

To provide an accessible interface in a logically concise manner, we develop an online application based on the DeepTrio model. The PPI prediction results and *importance maps* can be easily obtained by submitting two protein sequences to the web server. Moreover, the results from multiple submissions will be recorded on the web page, and they can be conveniently filtered and downloaded from the website. This online application is available at http://bis.zju.edu.cn/deeptrio.

## 4 Conclusion

With the development of deep-learning algorithms such as CNN (LeCun and Bengio, 1995), recurrent neural networks (Hochreiter and Schmidhuber, 1997) and graph neural networks (Scarselli *et al.*, 2009), an increasing number of sequence-based deep-learning methods have been developed for PPI prediction. A state-of-the-art approach, PIPR, adopts an RCNN architecture to capture the local features and contextualized information and has achieved remarkable performance, whereas it does not provide a convenient implementation for inexperienced users and a visualization method to make the model interpretable. However, DeepTrio provides a superior prediction for PPI and an intuitive visualization for the importance of each protein residue in both online and offline implements. Besides, a variety of experimental evaluations show that the additional single-protein training indeed improves the performance of PPI prediction by inherently preventing weight polarization. For future work, a possible direction is to incorporate molecular docking calculation into DeepTrio for more accurate prediction of key regions for PPI. We also explore the possibilities of using dynamic visualization techniques to interpret our model better.

In summary, we propose a deep-learning-based model, DeepTrio, to predict PPIs using raw protein sequences. By adopting the multiple parallel convolution filter architecture that allows DeepTrio to capture the deep features from the protein profiles, our model achieves encouraging performance on the benchmark datasets in terms of various evaluation metrics. We also integrate the single-protein training strategy and masking operation to prevent weight polarization in the intermediary layers and enable DeepTrio to visualize the contribution of each protein residue to the prediction results. Furthermore, we also provide an online application for PPI prediction and important residue detection.

## Funding

This work was supported by the National Key Research and Development Program of China [2016YFA0501704, 2018YFC0310602]; the National Natural Sciences Foundation of China [31771477, 32070677]; the 151 Talent Project of Zhejiang Province (first level); Jiangsu Collaborative Innovation Center for Modern Crop Production and Collaborative Innovation Center for Modern Crop Production cosponsored by province and ministry.

*Conflict of Interest*: none declared. 

## Supplementary Material

btab737_Supplementary_Data
