# Supplementary material for: DeepTrio: a ternary prediction system for protein–protein interaction using mask multiple parallel convolutional neural networks
Source: Bioinformatics. 2021 Oct 25;38(3):694–702. doi: 10.1093/bioinformatics/btab737 (PMC8756175; doi:10.1093/bioinformatics/btab737)
Supplement: btab737_Supplementary_Data [file btab737_supplementary_data.docx]

**Supplementary Materials**

## 1 Hyper-parameter tuning

We examine thirteen critical hyper-parameters (Table S1) of the DeepTrio model, which may affect the prediction performance of our model. The hyper-parameter searching process is implemented by GpyOpt tool on the BioGRID yeast multi-validated physical interaction dataset. We set the number of initial random searching points to 10, and run for a maximum of 50 epochs, and finally get 32 acquisitions. The performance of all candidate models and their corresponding hyper-parameter settings are shown in Supplementary Table S2.

**Table S1.** Thirteen hyper-parameters for DeepTrio and their domains

| No. | Hyper-parameter | Values | Description |
| --- | --- | --- | --- |
| 1 | Embedding dimension | {10, 15, 20} |  |
| 2 | The spatial dropout rate of embedding outputs | {0.005, 0.01} |  |
| 3 | The length coefficient of small-size convolution kernels | {0.12, 0.14, 0.16} | specifying the length of the small-size convolution windows |
| 4 | The stride coefficient of small-size convolution kernels | {0.15, 0.2, 0.25} | specifying the stride length of the small-size convolution kernels |
| 5 | The length coefficient of large-size convolution kernels | {0.1, 0.12, 0.14} | specifying the length of the large-size convolution windows |
| 6 | The stride coefficient of large-size convolution kernels | {0.25, 0.3, 0.35} | specifying the stride length of the large-size convolution kernels |
| 7 | The number of small-size convolution kernels | {100, 125, 150} | specifying the number of convolution kernels in each small-size filter |
| 8 | The number of large-size convolution kernels | {100, 125, 150} | specifying the number of convolution kernels in each large-size filter |
| 9 | The spatial dropout rate of convolution outputs | {0.05, 0.1, 0.15} |  |
| 10 | Fully connected layer dimension | {128, 256} |  |
| 11 | The dropout rate of the first dense layer | {0.1, 0.2} |  |
| 12 | The dropout rate of the second dense layer | {0.1, 0.2} |  |
| 13 | Optimizer | {Adam, AMSGrad} |  |

**Table S2.** Performance comparison of DeepTrio candidate models with different hyper-parameters

| **Arg.**  **Candidate** | **1** | **2** | **3** | **4** | **5** | **6** | **7** | **8** | **9** | **10** | **11** | **12** | **13** | **Accuracy** |
| --- | --- | --- | --- | --- | --- | --- | --- | --- | --- | --- | --- | --- | --- | --- |
| 1 | 20 | 0.01 | 0.16 | 0.15 | 0.12 | 0.25 | 150 | 175 | 0.05 | 0.2 | 0.1 | 128 | Yes | 0.9755 |
| 2 | 20 | 0.01 | 0.12 | 0.2 | 0.1 | 0.3 | 150 | 150 | 0.15 | 0.1 | 0.1 | 256 | Yes | 0.9783 |
| 3 | 20 | 0.01 | 0.14 | 0.15 | 0.12 | 0.25 | 125 | 175 | 0.1 | 0.1 | 0.1 | 128 | No | 0.9766 |
| 4 | 20 | 0.01 | 0.14 | 0.15 | 0.12 | 0.35 | 100 | 175 | 0.1 | 0.1 | 0.1 | 256 | No | 0.9731 |
| 5 | 20 | 0.01 | 0.16 | 0.2 | 0.1 | 0.35 | 150 | 150 | 0.15 | 0.1 | 0.1 | 128 | Yes | 0.9749 |
| 6 | 20 | 0.01 | 0.12 | 0.15 | 0.12 | 0.25 | 150 | 150 | 0.1 | 0.1 | 0.1 | 256 | Yes | 0.9779 |
| 7 | 20 | 0.005 | 0.16 | 0.25 | 0.12 | 0.35 | 100 | 150 | 0.05 | 0.2 | 0.1 | 256 | Yes | 0.9740 |
| 8 | 20 | 0.005 | 0.16 | 0.15 | 0.14 | 0.25 | 150 | 175 | 0.05 | 0.2 | 0.1 | 128 | Yes | 0.9788 |
| 9 | 20 | 0.005 | 0.16 | 0.15 | 0.14 | 0.25 | 150 | 175 | 0.05 | 0.2 | 0.1 | 256 | Yes | 0.9775 |
| 10 | 15 | 0.01 | 0.14 | 0.25 | 0.14 | 0.25 | 100 | 200 | 0.1 | 0.2 | 0.2 | 256 | Yes | 0.9755 |
| 11 | 15 | 0.01 | 0.16 | 0.15 | 0.14 | 0.25 | 150 | 175 | 0.15 | 0.2 | 0.1 | 128 | Yes | 0.97493 |
| 12 | 15 | 0.01 | 0.16 | 0.2 | 0.1 | 0.3 | 150 | 175 | 0.1 | 0.2 | 0.2 | 256 | Yes | 0.9733 |
| 13 | 15 | 0.01 | 0.16 | 0.25 | 0.14 | 0.35 | 150 | 175 | 0.15 | 0.2 | 0.2 | 256 | Yes | 0.9760 |
| 14 | 15 | 0.01 | 0.14 | 0.15 | 0.14 | 0.3 | 150 | 150 | 0.15 | 0.1 | 0.2 | 256 | Yes | 0.9777 |
| 15 | 15 | 0.005 | 0.14 | 0.2 | 0.1 | 0.3 | 125 | 175 | 0.05 | 0.2 | 0.1 | 128 | No | 0.9741 |
| 16 | 15 | 0.005 | 0.14 | 0.2 | 0.1 | 0.3 | 125 | 175 | 0.05 | 0.2 | 0.1 | 128 | Yes | 0.9784 |
| 17 | 15 | 0.005 | 0.12 | 0.15 | 0.1 | 0.25 | 150 | 175 | 0.05 | 0.2 | 0.1 | 128 | No | 0.9721 |
| 18 | 15 | 0.005 | 0.12 | 0.15 | 0.1 | 0.25 | 150 | 175 | 0.05 | 0.2 | 0.1 | 128 | Yes | 0.9734 |
| 19 | 15 | 0.005 | 0.12 | 0.15 | 0.1 | 0.25 | 150 | 175 | 0.05 | 0.2 | 0.1 | 256 | Yes | 0.9783 |
| 20 | 15 | 0.005 | 0.16 | 0.15 | 0.14 | 0.25 | 150 | 175 | 0.05 | 0.2 | 0.1 | 128 | Yes | 0.97883 |
| 21 | 15 | 0.005 | 0.12 | 0.2 | 0.14 | 0.3 | 150 | 175 | 0.15 | 0.2 | 0.1 | 256 | Yes | 0.9755 |
| 22 | 15 | 0.005 | 0.16 | 0.15 | 0.14 | 0.25 | 150 | 175 | 0.05 | 0.2 | 0.1 | 128 | No | 0.97512 |
| **23** | **15** | **0.005** | **0.16** | **0.15** | **0.14** | **0.25** | **150** | **175** | **0.05** | **0.2** | **0.1** | **256** | **Yes** | **0.9796** |
| 24 | 15 | 0.005 | 0.16 | 0.25 | 0.14 | 0.35 | 125 | 175 | 0.1 | 0.1 | 0.1 | 128 | Yes | 0.9755 |
| 25 | 15 | 0.005 | 0.14 | 0.25 | 0.14 | 0.35 | 150 | 200 | 0.1 | 0.1 | 0.1 | 256 | Yes | 0.97400 |
| 26 | 15 | 0.005 | 0.12 | 0.25 | 0.14 | 0.35 | 150 | 150 | 0.1 | 0.1 | 0.2 | 128 | Yes | 0.97809 |
| 27 | 15 | 0.005 | 0.12 | 0.15 | 0.14 | 0.35 | 150 | 150 | 0.1 | 0.1 | 0.2 | 128 | No | 0.9749 |
| 28 | 15 | 0.005 | 0.14 | 0.25 | 0.14 | 0.3 | 100 | 175 | 0.15 | 0.1 | 0.2 | 256 | No | 0.9708 |
| 29 | 15 | 0.005 | 0.12 | 0.2 | 0.14 | 0.3 | 125 | 150 | 0.05 | 0.1 | 0.1 | 128 | No | 0.97418 |
| 30 | 10 | 0.01 | 0.14 | 0.15 | 0.1 | 0.35 | 125 | 150 | 0.15 | 0.2 | 0.2 | 256 | No | 0.9688 |
| 31 | 10 | 0.01 | 0.16 | 0.25 | 0.14 | 0.35 | 150 | 150 | 0.15 | 0.1 | 0.2 | 128 | Yes | 0.97623 |
| 32 | 10 | 0.01 | 0.16 | 0.25 | 0.14 | 0.25 | 100 | 175 | 0.1 | 0.1 | 0.1 | 128 | No | 0.97177 |

*Note:* Bold font stands for the optimal hyper-parameter combination for the best model performance. Arguments 1-13 correspond to thirteen hyper-parameters in Table S1.

**Table S3.** Details of the performance of DeepTrio and PIPR on *S.cerevisiae* core dataset from DeepFE-PPI

| Turn | Accuracy | Precision | Sensitivity | Specificity | F1-score | MCC | AUC |
| --- | --- | --- | --- | --- | --- | --- | --- |
| DeepTrio | | | | | | | |
| 1 | 0.935009488 | 0.959835220 | 0.90485437 | 0.963821891 | 0.93153423 | 0.871156334 | 0.965410595 |
| 2 | 0.930740038 | 0.980146289 | 0.88075117 | 0.981783316 | 0.92779426 | 0.866163339 | 0.968425767 |
| 3 | 0.918367347 | 0.963427376 | 0.87063267 | 0.966603053 | 0.91468254 | 0.840758921 | 0.959176704 |
| 4 | 0.920265781 | 0.957202504 | 0.87835249 | 0.961429914 | 0.91608392 | 0.843219972 | 0.961099667 |
| 5 | 0.924062648 | 0.956043955 | 0.89189189 | 0.957446808 | 0.92285439 | 0.850249610 | 0.954789713 |
| PIPR | | | | | | | |
| 1 | 0.930740038 | 0.952748312 | 0.91059908 | 0.952101661 | 0.93119698 | 0.862439706 | 0.973684969 |
| 2 | 0.923588040 | 0.941591137 | 0.90077071 | 0.945743685 | 0.92072870 | 0.847821956 | 0.968729441 |
| 3 | 0.920740389 | 0.943031535 | 0.89306358 | 0.947614592 | 0.91736764 | 0.842475711 | 0.968512250 |
| 4 | 0.918367347 | 0.937621831 | 0.89906542 | 0.938283509 | 0.91793893 | 0.837531615 | 0.970725223 |
| 5 | 0.919791172 | 0.933266931 | 0.90182868 | 0.937265917 | 0.91727851 | 0.839942803 | 0.963761161 |

**Table S4.** Details of the performance of DeepTrio and DeepFE-PPI on *S.cerevisiae* core dataset from PIPR

| Turn | Accuracy | Precision | Sensitivity | Specificity | F1-score | MCC | AUC |
| --- | --- | --- | --- | --- | --- | --- | --- |
| DeepTrio | | | | | | | |
| 1 | 0.951074531 | 0.972061656 | 0.928242869 | 0.973636363 | 0.949647058 | 0.903027156 | 0.989128543 |
| 2 | 0.948331047 | 0.973658536 | 0.920664206 | 0.975521305 | 0.946420104 | 0.897915192 | 0.980889506 |
| 3 | 0.942844079 | 0.969844357 | 0.913840512 | 0.971715328 | 0.941009910 | 0.887146471 | 0.989526116 |
| 4 | 0.946935041 | 0.967711300 | 0.925522252 | 0.968663594 | 0.946146703 | 0.894761275 | 0.987795762 |
| 5 | 0.949679780 | 0.975704567 | 0.921946739 | 0.977210573 | 0.948064211 | 0.900696693 | 0.987933533 |
| DeepFE-PPI | | | | | | | |
| 1 | 0.912665752 | 0.905575539 | 0.921317474 | 0.904021937 | 0.913378684 | 0.825457468 | 0.950002174 |
| 2 | 0.913123000 | 0.914678898 | 0.911334551 | 0.914913082 | 0.913003663 | 0.826251781 | 0.938898609 |
| 3 | 0.910836763 | 0.879324894 | 0.952468006 | 0.869167428 | 0.914436156 | 0.824526974 | 0.959618379 |
| 4 | 0.913540714 | 0.883050847 | 0.953339432 | 0.873741994 | 0.916849978 | 0.829714035 | 0.962961924 |
| 5 | 0.901646844 | 0.874573378 | 0.937785909 | 0.865507776 | 0.905077262 | 0.805400199 | 0.953981044 |

## 2 Data availability

BioGRID human and yeast multi-validated physical interaction datasets and the *S.cerevisiae* core datasets from PIPR and DeepFE-PPI are available at https://github.com/huxiaoti/deeptrio.
